# Elevating Circulating L‐Kynurenine Promotes Frailty in Aging Mice

**DOI:** 10.1002/jcsm.70214

**Published:** 2026-02-02

**Authors:** Mia Y. Kawaida, Abigail L. Tice, Samuel Alvarez, Jacob A. Lackey, Benjamin Izaguirre, Qingping Yang, Lan Wei‐LaPierre, Russell T. Hepple, Terence E. Ryan

**Affiliations:** ^1^ Department of Applied Physiology and Kinesiology The University of Florida Gainesville Florida USA; ^2^ Center for Exercise Science The University of Florida Gainesville Florida USA; ^3^ Myology Institute The University of Florida Gainesville Florida USA; ^4^ Department of Physical Therapy The University of Florida Gainesville Florida USA

**Keywords:** aging, frailty, mitochondria, muscle, physical function

## Abstract

**Background:**

L‐Kynurenine (L‐Kyn), a product of tryptophan catabolism, increases with age and has been associated with reduced physical function and increased frailty in humans. Robustly expressed in skeletal muscle, kynurenine aminotransferases (KATs) degrade L‐Kyn into kynurenic acid and are regulated by the transcriptional co‐regulator peroxisome proliferator–activated receptor gamma coactivator 1‐alpha (PGC1α).

**Methods:**

The study investigated (1) if elevating L‐Kyn levels via a diet intervention exacerbates an age‐related decline in physical, muscle and mitochondrial functions and (2) if transgenic expression of PGC1α in skeletal muscle (MCK‐PGC1α) protects against age‐dependent L‐Kyn associated pathology in a cohort of aging MCK‐PGC1α transgenic mice and their wildtype littermates of both sexes (*n* = 262). Physical function was assessed longitudinally from 16 to 24 months of age using treadmill endurance capacity, grip strength, walking speed and daily physical activity. Muscle function was assessed in situ using nerve‐mediated contraction of the soleus muscle. Mitochondrial energetics were assessed using high resolution respirometry and fluorescence spectroscopy.

**Results:**

MCK‐PGC1α transgenic mice had significantly higher KAT expression ~2–5‐fold compared with wildtype littermates (*p* < 0.0001 for all isoforms). A main effect of L‐Kyn diet was observed for decreasing treadmill endurance capacity and daily physical activity in male mice (*p* ≦ 0.002). A main effect of L‐Kyn diet for decreasing maximal walking speed only was found in female mice (*p* = 0.037). Correspondingly, L‐Kyn increased frailty prevalence in male (+17%) and female (+26%) wildtype mice (*p* = 0.025 and 0.0001 respectively), which was mitigated by MCK‐PGC1α in both sexes. Soleus muscle strength and power were not impacted by diet or genotype in either sex (*p* > 0.5). Mitochondrial oxidative phosphorylation function in male and female MCK‐PGC1α mice was greater than wild type mice regardless of diet (*p* < 0.04), which is likely driven by upregulated expression of mitochondrial biogenesis related genes.

**Conclusions:**

We conclude that PGC1α overexpression in skeletal muscle mitigates the exacerbation of physical frailty induced by elevated circulating L‐Kyn in aging mice, in part through increased skeletal muscle capacity for L‐Kyn metabolism due to PGC1α‐induced increase in muscle KAT expression.

**Abbreviations**
AUR: Amplex UltraRed
Cr: creatine
CSA: cross‐sectional area
IDO: indoleamine 2, 3‐dioxygenase 1
KAT: kynurenine aminotransferase
L‐Kyn: L‐Kynurenine
MCK: muscle creatine kinase
MIM: mitochondrial isolation medium
MUNE: motor unit number estimation
OXPHOS: oxidative phosphorylation
PCr: phosphocreatine
PGC1α: peroxisome proliferator‐activated receptor gamma coactivator 1‐alpha
TMRM: tetramethylrhodamine methyl ester
TDO: tryptophan 2, 3‐dioxygenase
Trp: tryptophan

## 1 Introduction

Aging is associated with the deterioration of metabolic, physical and physiological functions that result in a decline in health and mobility. Aging results in a progressive decrease in skeletal muscle mass and strength in both men and women [1]. Frailty is a multidimensional state of increased vulnerability resulting from the age‐associated declines in physiological functions and is most often quantified via physical performance and body weight/composition [2, 3]. Estimates suggest that 12%–20% of adults over the age of 50 have frailty [4], a number that is expected to increase as the proportion of the global population in this age range grows. Recent human studies have reported that alterations in circulating tryptophan (Trp) metabolites, including an increase in L‐Kynurenine (L‐Kyn) levels, strongly associate with physical performance, frailty and skeletal muscle mass [5, 6, 7, 8, 9]. L‐Kyn is a product of Trp catabolism by indoleamine 2,3‐dioxygenase or tryptophan 2,3‐dioxygenase, and the ratio of circulating L‐Kyn to Trp (L‐Kyn/Trp) distinguishes low and high‐functioning octogenarians [10]. Beyond skeletal muscle, L‐Kyn accumulation has been shown to influence multiple physiological processes including altered neurotransmitters in the central nervous system [11], dysregulated hepatic metabolism [12] and impaired adaptive immunity [13], all of which may contribute to declines in physical function associated with L‐Kyn accumulation. Despite these associations, a causal relationship between increased circulating L‐Kyn and age‐associated declines in physical function or increases in frailty has not been tested.

Skeletal muscle, due to its large mass which declines with aging, may play a role in both the accumulation and catabolism of L‐Kyn. Kynurenine aminotransferases (KATs), of which two isoforms are located in the cytosol (KAT1/CCBL1 and KAT2/AADAT) and two within the mitochondria (KAT3/CCBL2 and KAT4/GOT2), convert L‐Kyn into kynurenic acid. Alternatively, kynurenine 3‐monooxygenase and kynureninase can convert L‐Kyn into 3‐hydroxykynurenine and anthranilic acid, respectively, which are precursors for the formation of nicotinamide adenine dinucleotide (NAD^+^) and quinolinic acid. Whereas NAD^+^ is an essential component of energy metabolism pathways and its homeostasis is compromised with advancing age, quinolinic acid is neurotoxic [5]. Considering that the mitochondrion is a site for L‐Kyn degradation and mitochondrial dysfunction is a hallmark of muscle aging [14], there may be a feed‐forward mechanism by which L‐Kyn promotes declines in muscle mass [15] and mitochondrial function [16] that reduce the capacity for L‐Kyn catabolism, thus promoting its accumulation. KAT expression is controlled by the transcriptional co‐regulator peroxisome proliferator–activated receptor gamma, coactivator 1 alpha (PGC1α) [17, 18], which is expressed at higher levels in oxidative muscle fibres [19, 20, 21] and induced by exercise [22]. Exercise remains one of the most successful strategies to slow skeletal muscle aging phenotypes, and highly functioning octogenarians display higher levels of KAT3 and KAT4 abundance and lower L‐Kyn/Trp ratio compared with lower functioning octogenarians [10].

Herein, we tested if increased circulating L‐Kyn directly exacerbates the age‐associated decline in physical function and increases the prevalence of frailty. To elevate L‐Kyn levels at earlier ages, we employed a L‐Kyn‐supplemented diet model [16]. Additionally, we employed transgenic expression of PGC1α specifically in skeletal muscle (MCK‐PGC1α) to test whether enhancing skeletal muscle L‐Kyn metabolism could reduce the age‐associated declines in physical function and the prevalence of frailty in aging mice.

## 2 Methods

### 2.1 Animals and Diet Intervention

All animal procedures were approved by the Institutional Animal Care and Use Committee at the University of Florida (protocol 202 400 000 430). MCK‐PGC1α transgenic mice [20] were established using breeders obtained from Jackson Laboratories (stock no. 008231) by mating hemizygous MCK‐PGC1α with wildtype (WT) C57BL/6J (stock no. 000664). Hemizygous offspring with transgenic overexpression of MCK‐PGC1α and WT offspring lacking the transgene were used. Mice were housed in a temperature (22°C) controlled room with 12‐h light/dark cycles and ad libitum access to food and water. From birth until the age of 16 months, mice received a standard chow diet (Envigo Teklad Global 18% Protein Rodent Diet 2918 irradiated pellet). Following baseline physical function assessments, mice were randomised to receive the standard chow diet or a L‐Kyn supplemented diet (Envigo Teklad Global 18% Protein Rodent Diet 2918 irradiated pellet supplemented with 150 mg L‐Kyn per kilogram diet), with both diets provided ad libitum.

### 2.2 Physical Function and Frailty Assessments

A frailty assessment developed for mice [23, 24] was performed at baseline (16 months old) prior to diet randomisation and was repeated at 20 and 24 months of age. This assessment included analyses of body weight and composition, grip strength, walking speed, treadmill endurance capacity and voluntary wheel activity. Detailed descriptions of the assessments are provided in the Supporting Information. Frailty status was assigned as follows: For physical function outcomes (running capacity, walking speed, grip strength, cage activity), a lower cutoff value of the 20th percentile was employed. For body weight, criterion values below the 20th percentile (below average body weights) and above the 80th percentile (high body weights) were used. Mice were categorised as *pre‐frail* if one or two of the frailty indices met the above criteria or *frail* if three or more of the frailty indices met the criteria. Mice that met none of the criterion scores were identified as *non‐frail*.

### 2.3 Sarcopenic Status

We used a modified sarcopenia status assessment recently developed for mice [25]. Criteria used for the analysis included grip strength, hindlimb muscle mass and running capacity (measured at 24 months old) with cutoff values set at two standard deviations below the mean of 14‐month‐old mice of the same sex. Mice that did not meet any of the criteria were classified as *normal*, whereas mice that met one criterion were classified as *probable sarcopenia*, and two or more criteria were classified as having *sarcopenia*.

### 2.4 Other Outcome Measures

A detailed description of the of the following outcome measures are provided in the Supporting Information: (1) Plasma L‐Kyn and Tryp Quantification; (2) In situ Muscle Function; (3) Muscle Histology; (4) Mitochondrial Isolation, High‐Resolution Respirometry, Membrane Potential, H_2_O_2_ Production; (5) RNA Isolation and Quantitative PCR; (6) Serum Cytokine/chemokine Analysis.

### 2.5 Statistical Analysis

An a priori power analysis was performed using estimated variance of the frailty index outcome in aged mice as the primary outcome measure using values from previous publications [23, 24, 26] (partial *η* ^2^ = 0.60). We also considered historical data on the survival rates of C57BL6 mice, and our initial sample size aimed to account for an expected 50% attrition rate. A sample size of *n* = 14/group/sex was expected to give at least 80% power to detect a 15% difference in frailty (alpha = 0.05). All data are presented as mean ± standard deviation (SD). Normality was determined by the Shapiro–Wilk test. Data with normal distribution were analysed using two‐way or a mixed model analysis with Tukey's post hoc multiple comparisons. Pearson correlations were performed using two‐tailed testing. Differences in the prevalence of frailty and sarcopenia were analysed using a chi‐square test. Statistical analyses were performed using the GraphPad Prism software (version 10.3.0). Statistical significance was declared at *p* < 0.05.

## 3 Results

### 3.1 Establishing a Model to Causally Test the Role of Kynurenine Accumulation in the Decline of Physical Function With Aging

**FIGURE 1:**
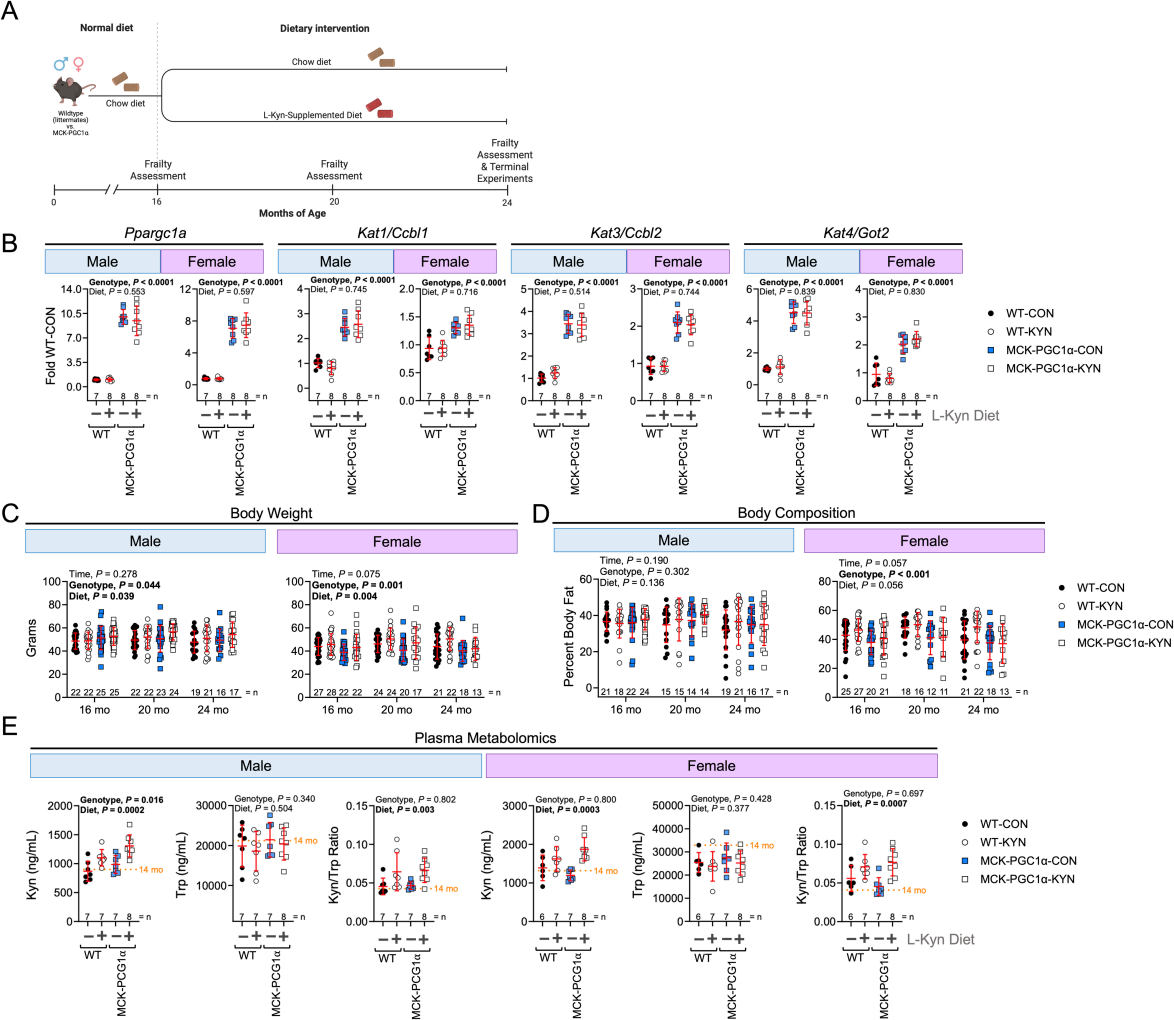
Consumption of a L‐Kyn supplemented diet raises circulating kynurenine levels, and MCK‐PGC1α expression increases expression of kynurenine aminotransferases. (A) Graphical depiction of the experimental timeline. (B) qPCR analysis showing gene expression in the gastrocnemius muscle. (C) Body weight. (D) Percent body fat measured by time‐domain nuclear magnetic resonance. (E) Plasma metabolomics quantification of L‐Kyn, tryptophan (Trp), and the L‐Kyn/Trp ratio. Panels B and E were analysed using two‐way ANOVA. Panels C and D were analysed using a mixed‐model. Mean values obtained from 14‐month‐old C57BL6 (*n* = 10/sex) obtained from the National Institute of Aging colony are shown for comparison. Data are presented as mean ± standard deviation.

To test if elevated kynurenine levels causally contribute to age‐related declines in physical function, we fed 16‐month WT littermates and MCK‐PGC1α transgenic mice a standard chow diet or a chow diet supplemented with 150 mg/kg L‐Kyn until 24 months of age (Figure 1A) as previously described [16]. MCK‐PGC1α mice were chosen because they express higher levels of *Kat1, Kat3* and *Kat4/Got2* in their skeletal muscle (Figure 1B), which improves kynurenine clearance [17, 27]. No differences were observed in food consumption between chow and L‐Kyn supplemented diets (Figure S1). A significant main effect for diet was detected for body weight in both male and female mice (Figure 1C), indicating that increasing circulating L‐Kyn induced a modest but significant level of weight gain compared with chow fed mice. There was also a main effect of genotype on body weight, with male MCK‐PGC1α mice having slightly higher body weights compared with WT littermates, but female MCK‐PGC1α mice having lower body weights compared with WT (*p* = 0.044 and *p* = 0.001 respectively). Body composition was not impacted by L‐Kyn diet in either sex; however, female MCK‐PGC1α mice had lower body fat percentages compared with WT mice (Figure 1D). Targeted plasma metabolomics confirmed that mice consuming the L‐Kyn supplemented diet had significantly higher levels of circulating L‐Kyn and L‐Kyn/Trp ratio than those who consumed a chow diet (Figure 1E). The diet had no effect on circulating tryptophan levels (*p* = 0.50 and *p* = 0.37 for male and female mice).

### 3.2 Increasing Circulating L‐Kyn and MCK‐PCG1α Expression Alter Physical Function in an Age‐ and Sex‐Dependent Manner

**FIGURE 2:**
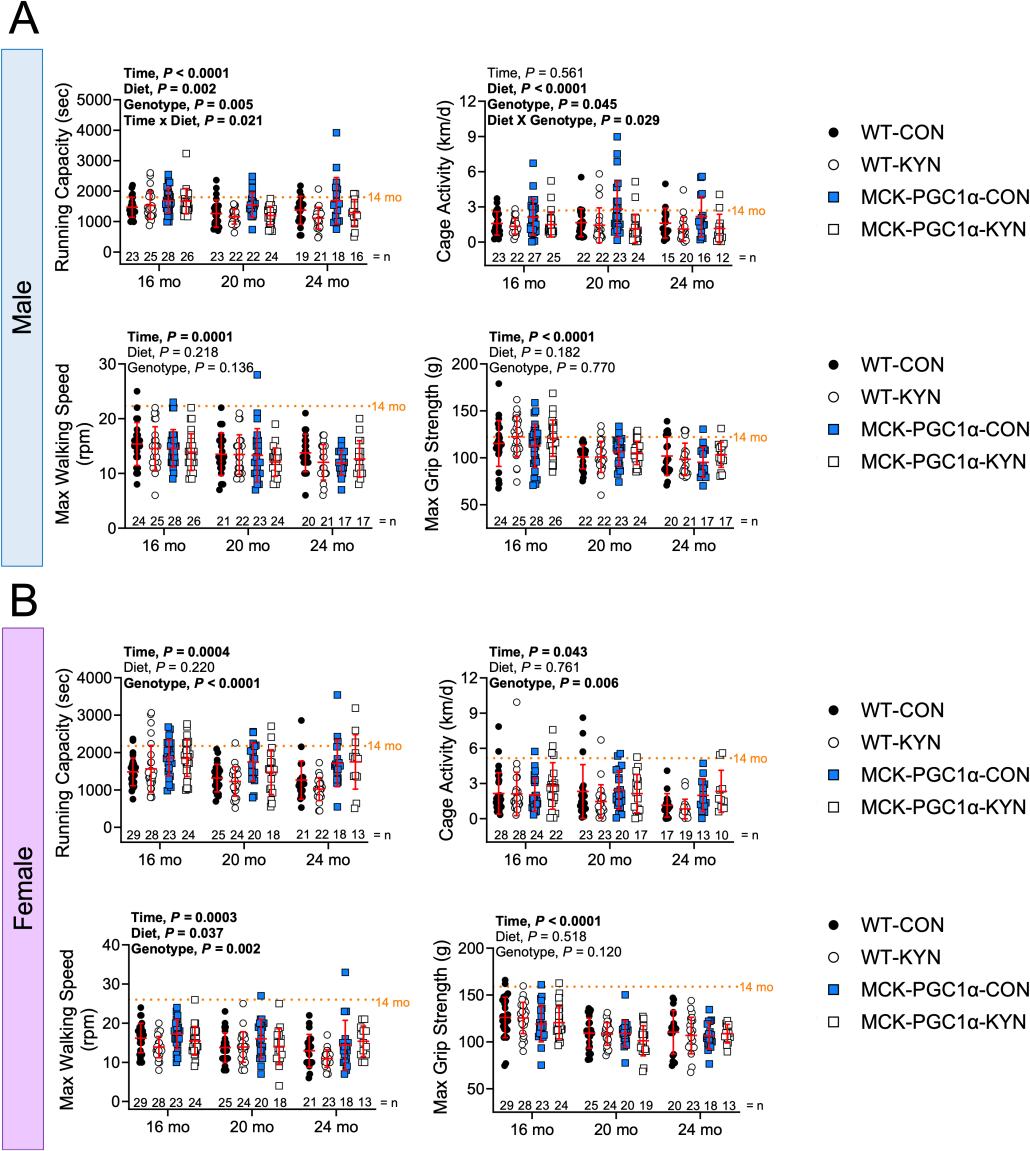
Increasing circulating L‐Kyn and MCK‐PCG1α expression alter physical function in an age‐ and sex‐dependent manner. (A) Physical function assessments including treadmill running capacity, cage wheel activity (average daily activity), walking speed (rotorod), and grip strength (forelimb) from male mice. (B) Physical function assessments including treadmill running capacity, cage wheel activity (average daily activity), walking speed (rotorod), and grip strength (forelimb) from female mice. Panels A and B were analysed using three‐way ANOVA. Mean values obtained from 14‐month‐old C57BL6 (*n* = 10/sex) obtained from the National Institute of Aging colony are shown for comparison. Data are presented as mean ± standard deviation.

To test whether elevated circulating L‐Kyn levels impact physical function in aging mice, we performed multiple assessments of physical function including running capacity, walking speed, grip strength and daily cage activity prior to randomising mice to either chow or L‐Kyn supplemented diets (16 months old). Mice were randomised to either chow or L‐Kyn diets to match baseline running capacity between groups. The physical function assessments were repeated at 16 (baseline), 20 and 24 months old. As expected, we observed nearly all physical function outcomes declined with age in both sexes (main effect of time, *p* < 0.05), except for daily cage activity in male mice (Figure 2A,B). In male mice, a significant diet effect was observed for running capacity (*p* = 0.002) and cage activity (*p* < 0.0001), indicating that elevated circulating L‐Kyn decreases running capacity and cage activity in male mice (Figure 2A). For significant interactions observed in males for running capacity and cage activity, post hoc pairwise comparisons are shown in Table S3. In female mice, a significant diet effect was only observed for walking speed (*p* = 0.037; Figure 2B). For benchmarking purposes, we also performed physical function analyses in 14‐month‐old C57BL/6 J male and female mice obtained from the National Institute of Aging colony. For all physical function outcomes, 24‐month‐old WT‐CON female mice display greater declines in running capacity (42%), walking speed (53%), grip strength (30%) and cage activity (77%) compared with values obtained from 14‐month‐old C57BL/6 J female mice. In comparison, male 24‐month‐old WT‐CON mice displayed substantially smaller declines in running capacity (23%), walking speed (37%), grip strength (17%) and cage activity (38%) compared with 14‐month‐old C57BL/6 J male mice.

MCK‐PGC1α transgenic mice have increased L‐Kyn biotransformation and reduced neurotoxicity [17, 18]. Additionally, studies in aged (24 months) mice with overexpression of PGC1α have reported resistance to muscle atrophy [28, 29]. Thus, we sought to determine if MCK‐PGC1α transgenic mice exhibit resistance to the negative effects of L‐Kyn supplemented diet. In male mice, a significant main effect of genotype was observed for running capacity (*p* = 0.005) and cage activity (*p* = 0.045; Figure 2A). However, a genotype × diet interaction was only detected for cage activity in male mice (*p* = 0.029). These results indicate that MCK‐PGC1α transgenic mice have higher running capacity and cage activities than their WT littermates but are similarly impacted by elevated L‐Kyn levels. In female mice, significant main effects of genotype were seen for running capacity (*p* < 0.0001), cage activity (*p* = 0.006) and walking speed (*p* = 0.002; Figure 2B), demonstrating that MCK‐PGC1α female mice have better physical function compared with their WT counterparts.

### 3.3 Elevating Circulating L‐Kyn Levels Increased the Prevalence of Physical Frailty Which Can Be Reduced by MCK‐PCG1α Expression

**FIGURE 3:**
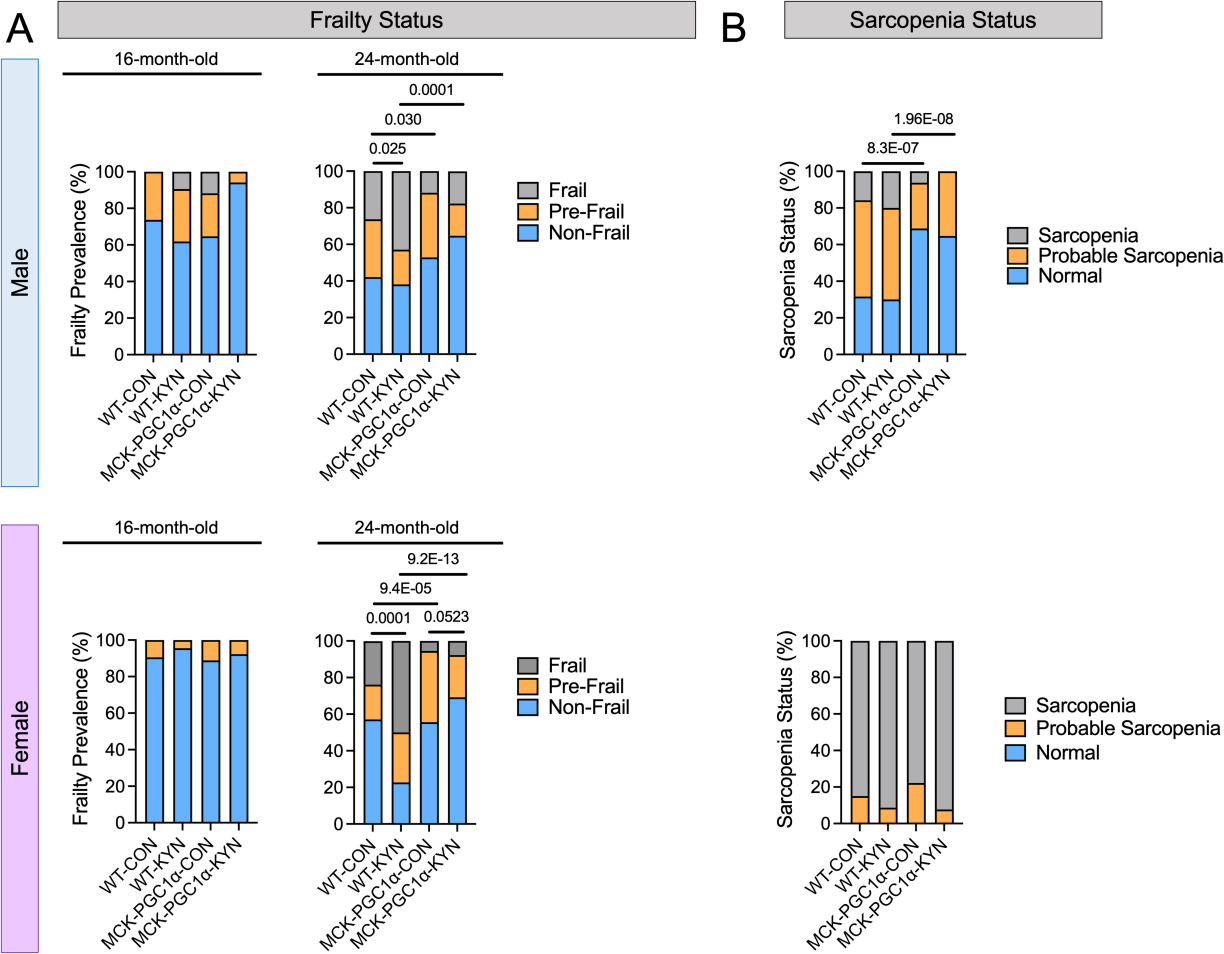
Elevating circulating L‐Kyn levels increased the prevalence of physical frailty, which can be reduced by MCK‐PCG1α expression. (A) Frailty status at 16 months old (prior to intervention) and 24 months old (after diet intervention) in male and female mice. (B) Sarcopenia status of mice at 24 months old using cutoff values obtained from 14‐month‐old adult controls of each sex. Data were analysed using chi‐squared test. Samples sizes are as follows: male WT‐CON (*n* = 20), male WT‐KYN (*n* = 21), male MCK‐PCG1α‐CON (*n* = 17), male MCK‐PCG1α‐KYN (*n* = 17), female WT‐CON (n = 20), female WT‐KYN (*n* = 23), female MCK‐PCG1α‐CON (*n* = 18), and female MCK‐PCG1α‐KYN (*n* = 13).

Because elevated L‐Kyn has been strongly linked to frailty in recent human studies [5, 6, 30], we utilised the physical function and body weight outcomes to assign a frailty status to mice. At 16 months old and prior to randomisation, there were no differences in the prevalence of frailty or pre‐frailty in any groups for both sexes (Figure 3A). The prevalence of frailty was significantly higher at 24 months old in WT male and female mice that consumed the L‐Kyn supplemented diet when compared with WT mice on the chow diet (*p* = 0.025 and *p* = 0.0001 for male and female respectively), confirming that elevated L‐Kyn levels increased the prevalence of frailty in aging mice. The prevalence of frailty and pre‐frailty was significantly lower in male and female MCK‐PGC1α mice under both chow and L‐Kyn diet conditions (Figure 3A), indicating that PGC1α overexpression in muscle reduces the likelihood of developing frailty in aging mice, potentially through enhanced muscle L‐Kyn catabolism.

Often, age‐related declines in muscle strength, muscle quantity/quality and physical performance are used to clinically diagnose sarcopenia [31]. Thus, we used a recently reported criterion for determining sarcopenic status [25]. In males, significantly more WT mice were classified with probable sarcopenia or sarcopenia compared with MCK‐PGC1α mice (Figure 3B), but no effect of L‐Kyn diet was observed. Strikingly, all 24‐month‐old female mice in our cohort were classified with probable sarcopenia or sarcopenia, but no effect of diet or genotype was seen (Figure 3B).

### 3.4 Contractile Properties of the Soleus in Situ Are Unaffected in Mice Consuming a L‐Kyn Supplemented Diet

**FIGURE 4:**
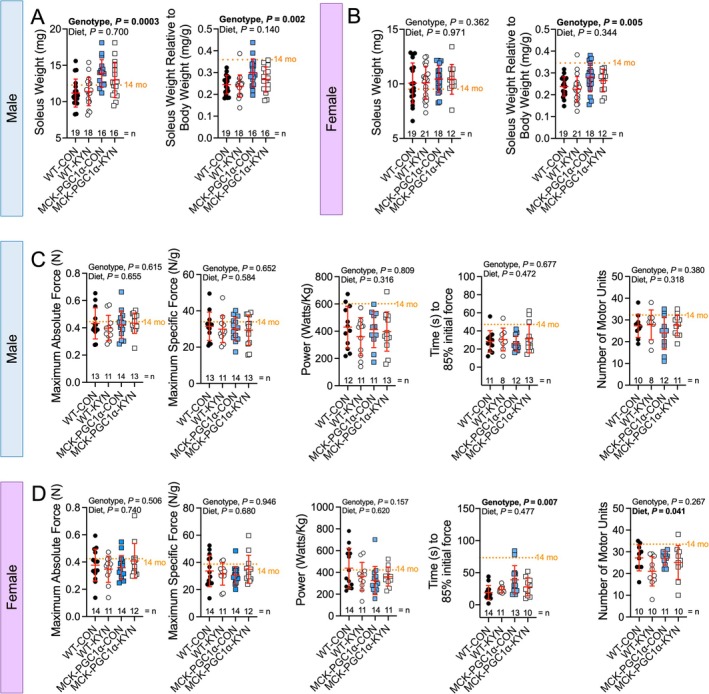
Contractile properties of the soleus in situ are unaffected in mice consuming a L‐Kyn supplemented diet. Soleus mass in absolute values and relative to body mass in (A) male and (B) female mice. (C) Maximum absolute and specific force, peak power at 35% of maximal force, muscle fatigue, and motor unit number estimation in male mice. (D) Maximum absolute and specific force, peak power at 35% of maximal force, muscle fatigue, and motor unit number estimation in female mice. Data were analysed using two‐way ANOVA. Mean values obtained from 14‐month‐old C57BL6 (*n* = 10/sex) obtained from the National Institute of Aging colony are shown for comparison. Data are presented as mean ± standard deviation.

Next, we assessed the active mechanical properties of the soleus muscle in situ prior to euthanasia. The soleus mass (relative to body weight) was larger in male (Figure 4A) and female (Figure 4B) MCK‐PGC1α mice but was not impacted by the L‐Kyn diet. Across numerous other skeletal muscles, MCK‐PGC1α mice displayed larger muscle masses compared with WT mice (Figure S2). To assess the contractile function of the soleus muscle, we measured maximal isometric force, peak power at 35% of the maximal force, fatiguability and a motor unit number estimation. In male mice, we did not detect effects of either the L‐Kyn diet or genotype on any of the muscle contractile function parameters (Figure 4C). In female mice, isometric force and peak power were unaffected by diet or genotype (Figure 4D); however, there was a significant genotype effect in muscle fatiguability with MCK‐PGC1α females having lower rates of force decline compared with WT mice (Figure 4D). A significant diet effect was also seen for the number of motor units in female mice (*p* = 0.041), demonstrating that L‐Kyn feeding reduced the number of motor units in females (Figure 4D). Motor unit number displayed strong positive correlations with peak absolute and specific force only in female mice (Figure S3).

### 3.5 Elevated Circulating L‐Kyn and MCK‐PGC1α Induce Histological Changes in the Aged Soleus Muscle

**FIGURE 5:**
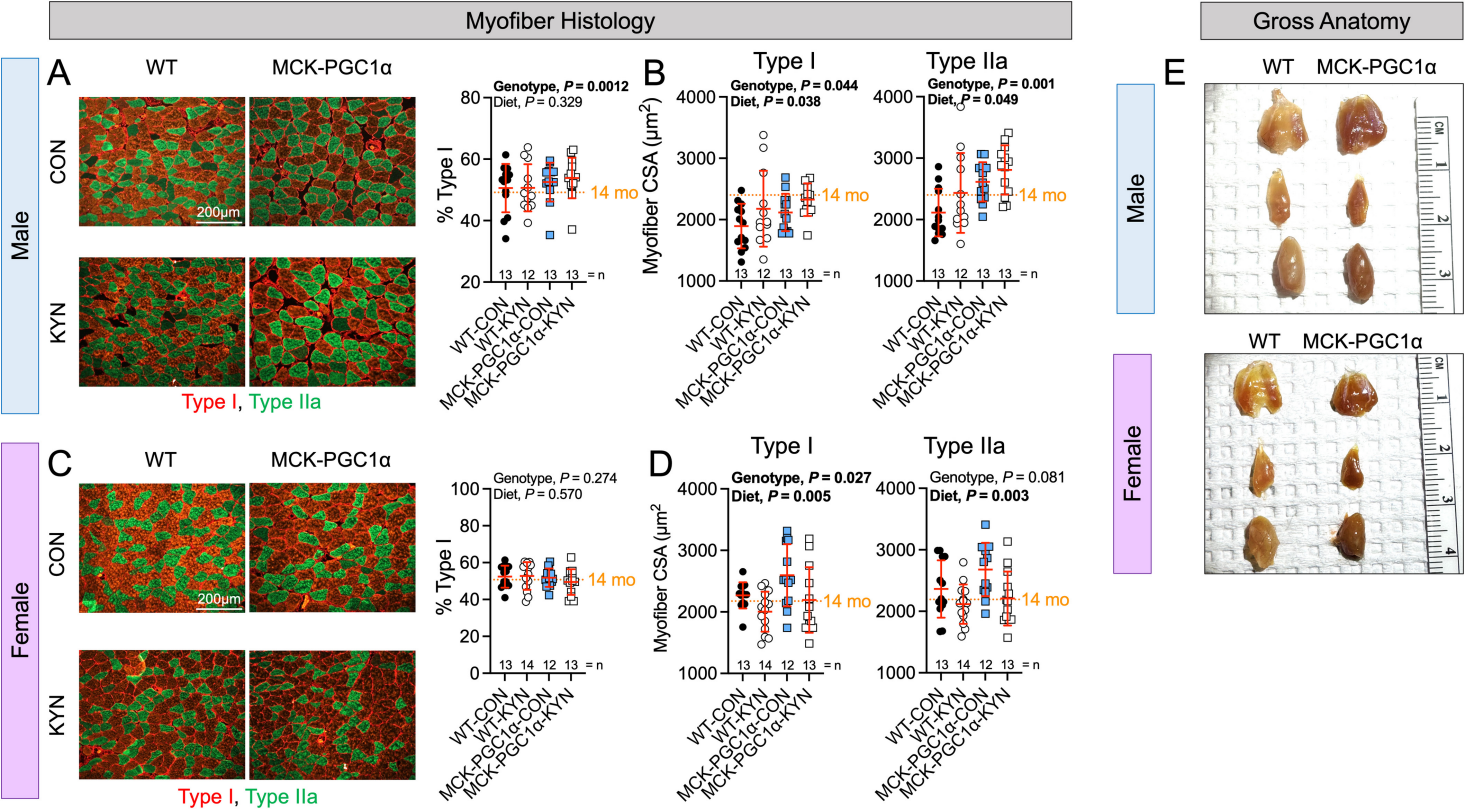
Elevated circulating L‐Kyn and MCK‐PGC1α induce histological changes in the aged soleus muscle. (A) Representative images of labelled cross sections of the soleus muscle and the percentage of type I myofibres in male mice. (B) Mean myofibre cross‐sectional area (CSA) of type I and IIa myofibres in male mice. (C) Representative images of labelled cross sections of the soleus muscle and the percentage of type I myofibres in female mice. (D) Mean myofibre CSA of type I and IIa myofibres in female mice. (E) Gross images of hindlimb muscles. Data were analysed using two‐way ANOVA. Mean values obtained from 14‐month‐old C57BL6 (*n* = 10/sex) obtained from the National Institute of Aging colony are shown for comparison. Data are presented as mean ± standard deviation.

Fibre typing analysis revealed that male MCK‐PGC1α had a greater percentage of slow twitch (Type I) myofibres compared with WT littermates (Figure 5A), a finding consistent with the literature [20]. The mean cross‐sectional area of Type I and Type IIa fibres was also significantly higher in male MCK‐PGC1α mice compared with male WT mice (Figure 5B). Unexpectedly, a significant main effect of diet was observed in male mice (Figure 5B) indicating that mice consuming the L‐Kyn supplemented diet had greater myofibre areas than those consuming the chow diet. In female mice, the proportion of Type I and Type IIa fibres was not impacted by diet or genotype (Figure 5C). The mean cross‐sectional area of Type I fibres (*p* = 0.0027) was also significantly higher in female MCK‐PGC1α mice compared with female WT mice, whereas the Type IIa fibres were statistically trending (*p* = 0.081) (Figure 5D). In contrast to males, female mice that consumed the L‐Kyn diet had significantly smaller cross‐sectional areas of Type I (*p* = 0.005) and Type IIa (*p* = 0.003) than mice that consumed the chow diet (Figure 5D). As expected, both male and female MCK‐PGC1α mice displayed a more red/oxidative appearance in their muscles (Figure 5E).

### 3.6 Elevated Circulating L‐Kyn and MCK‐PGC1α Alter Muscle Mitochondrial Energetics in Aging Mice

**FIGURE 6:**
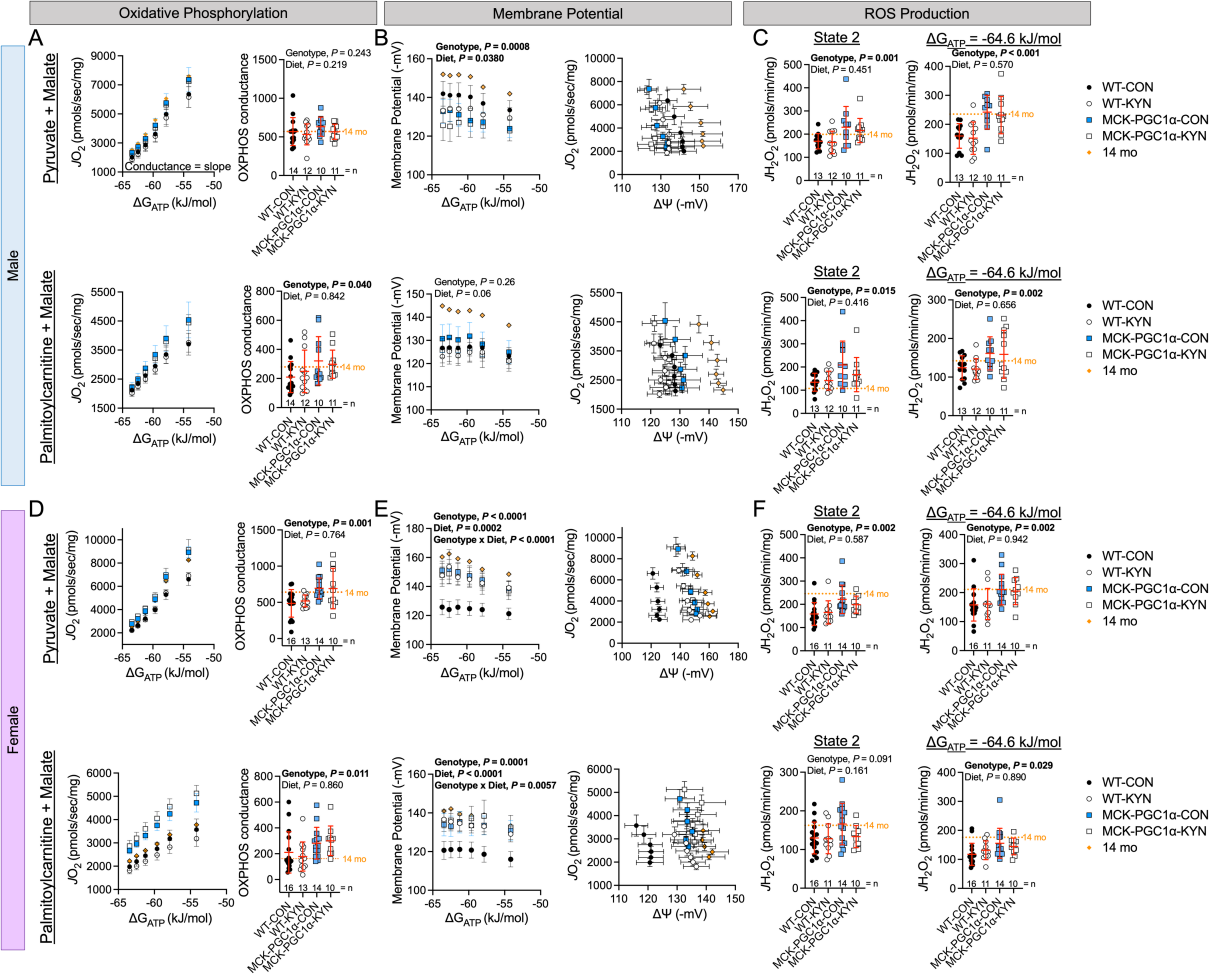
Elevated circulating L‐Kyn and MCK‐PGC1α alter muscle mitochondrial energetics in aging mice. (A) Mitochondrial OXPHOS function in male mice. (B) Mitochondrial membrane potential and respiratory efficiency in male mice. (C) Mitochondrial H_2_O_2_ emitting potential in male mice. (D) Mitochondrial OXPHOS function in female mice. (E) Mitochondrial membrane potential and respiratory efficiency in female mice. (F) Mitochondrial H_2_O_2_ emitting potential in female mice. Data were analysed using two‐way ANOVA. Mean values obtained from 14‐month‐old C57BL6 (*n* = 10/sex) obtained from the National Institute of Aging colony are shown for comparison. Data are presented as mean ± standard deviation.

In male mice, analysis of oxidative phosphorylation (OXPHOS) conductance (the linear relationship between *J*O_2_ and ΔG_ATP_) revealed that MCK‐PGC1α mice had greater OXPHOS conductance when fuelled with palmitoylcarnitine + malate only (Figure 6A), although the *J*O_2_ value at the highest energy demand assessed was greater in MCK‐PGC1α mice compared with WT littermates. Interestingly, MCK‐PGC1α male mice had lower membrane potentials when fuelled with pyruvate + malate only (Figure 6B). Mice that consumed the L‐Kyn supplemented diet had lower membrane potential compared with chow fed male mice (Figure 6B). Thus, examination of the relationship between *J*O_2_ and membrane potential, which provides an estimation of respiratory efficiency, shows that mice that consumed L‐Kyn diets had a leftward shift (Figure 6B) indicative of decreased mitochondrial respiratory efficiency in male mice. We also observed that male MCK‐PGC1α has greater H_2_O_2_ emitting potential under State 2 and resting energy demand conditions, but there was no effect of diet (Figure 6C).

Female MCK‐PGC1α mice had significantly higher OXPHOS conductance compared with their WT counterparts (Figure 6D). There was no effect of diet on OXPHOS conductance in female mice (Figure 6D). Female MCK‐PGC1α mice also had significantly greater membrane potentials compared with WT mice under both fuel conditions (Figure 6E). In contrast to male mice, female mice that consumed the L‐Kyn supplemented diet had greater membrane potentials compared with chow fed female mice in both fuel conditions (Figure 6B). A significant genotype × diet interaction was observed, indicating that L‐Kyn‐induced increased membrane potential was driven by WT female mice. Consequently, respiratory efficiency was lowest in WT‐CON females and significantly greater in MCK‐PGC1α (both diets) and WT‐KYN female mice (Figure 6E). Like males, female MCK‐PGC1α mice had significantly higher H_2_O_2_ emitting potential compared with WT mice, but no diet effect was observed (Figure 6F). These data revealed that elevated circulating L‐Kyn levels induce changes in skeletal muscle mitochondrial energy transduction in a sex‐specific manner.

### 3.7 Elevated L‐Kyn and Overexpression of MCK‐PGC1α Do Not Impact Circulating Inflammatory Markers

As previous studies have linked both L‐Kyn and aging to inflammation [8, 32], we performed a multiplex analysis of 25 cytokines and chemokines in serum collected at euthanasia. Most inflammatory markers were not altered by genotype or diet (Table S2). Exceptions include interleukin 7 (IL‐7), which was higher in male MCK‐PGC1α mice compared with WT mice regardless of diet (CON diet, *p* = 0.0331; KYN diet, *p* = 0.0511). IL‐10 was also different among groups but exhibited differing diet and genotype effects in each sex (Table S2). IL‐15 was higher in male MCK‐PGC1α mice compared with WT male mice. Finally, macrophage inflammatory protein 1 alpha and beta were significantly lower in female MCK‐PGC1α mice compared with WT female mice consuming the L‐Kyn diet.

## 4 Discussion

This is the first study to test a causal role of elevated circulating L‐Kyn levels on the development of frailty. Recent studies have reported higher serum L‐Kyn/Trp ratios in frail older adults [5, 7]. Moreover, serum L‐Kyn levels were positively correlated with the frailty index and negatively correlated with grip strength and gait speed even after adjusting for body mass index, age and sex. Ubaida‐Mohien et al. [10] demonstrated that a group of low‐functioning octogenarians had a higher L‐Kyn/Trp ratio than high‐functioning octogenarians. Herein, we observed that elevating circulating L‐Kyn levels in a cohort of aging mice exacerbated the age‐dependent decline in physical function and significantly increased the prevalence of frailty. Our findings are consistent with findings in Drosophila where elevating kynurenine pathway metabolites via the diet resulted in reduced climbing speeds, endurance and life span [33]. When inhibitors of the kynurenine pathway were given, flies had improved physical function and increased maximum life span [33]. In the current study, muscle‐specific overexpression of PGC1α enhanced the expression of KAT enzymes, which was associated with a significant reduction in the prevalence of frailty in both male and female mice. Given the pleiotropic effects of PGC1α overexpression in skeletal muscle, the current study cannot establish whether the beneficial effects observed are solely due to the impacts on L‐Kyn degradation. However, these findings establish kynurenine accumulation as a contributor to age‐related decline in physical function and frailty and indicate that interventions that enhance L‐Kyn catabolism may play a role in lessening the decline in physical function.

The underlying cause of increased circulating kynurenines in aging is not understood. Tryptophan is an essential amino acid metabolised primarily via the kynurenine pathway into a range of bioactive metabolites. Indoleamine 2, 3‐dioxygenase 1 (IDO) and tryptophan 2,3‐dioxygenase (TDO) are key enzymes that catalyse the degradation of tryptophan. Whereas IDO is expressed in multiple cell types and is upregulated during inflammation [5, 34], TDO is expressed primarily in the liver. Chronic inflammation has been strongly linked to the age‐associated decline in muscle mass and strength [32], and recent studies have implicated aberrant kynurenine pathway activity as a mediator of this relationship in aged men [8]. L‐Kyn can be transported into cells by large neutral amino acid transporters where KAT enzymes convert it to kynurenic acid [17, 35]. Skeletal muscle robustly expresses three KAT enzymes (KAT1/CCBL1, KAT3/CCBL2 and KAT4/GOT2) [19] and their expression increases with exercise [22, 36]. Considering aging is associated with a progressive loss of muscle mass, the accumulation of kynurenines in blood could be partially driven by a progressive decline in L‐Kyn degradation capacity by muscle. Together, it seems likely that the combination of increased IDO activity driven by inflammation and decline of peripheral kynurenine catabolism increase circulating kynurenines in aging. In support of this, high‐functioning octogenarians (masters athletes) have greater expression of KAT enzymes, lower inflammation and lower L‐Kyn/Trp ratios compared with low‐functioning octogenarians [10].

We employed an L‐Kyn supplemented diet model to chronically increase the circulating L‐Kyn level in mice beginning at the age of 16 months. This timepoint was chosen because it precedes the age at which physical frailty typically begins to manifest in mice [23, 24, 26]. In the WT mice, a higher prevalence of frailty and pre‐frailty was observed in the group that consumed the L‐Kyn supplemented diet. This effect was not observed in the MCK‐PGC1α mice. Interestingly, we employed a recent definition of sarcopenia in mice [25] and found no significant impact of L‐Kyn supplemented diet on sarcopenia in either sex. However, the prevalence of sarcopenia or probable sarcopenia was significantly lower in male MCK‐PGC1α mice compared with male WT mice. The lack of agreement between physical frailty status and sarcopenic status may be related to how comparisons are made. For frailty, mice were stratified based upon their physical function capability relative to the entire cohort (*n* = 206), whereas sarcopenic status requires comparisons to a small cohort of younger mice (*n* = 10) due to the terminal nature of measuring muscle mass. In assessing the outcome measures used to designate frailty status, having a low treadmill endurance capacity and low daily physical activity (cage wheel) appeared to best identify the most vulnerable mice that were more likely to experience early mortality or the most significant decreases in physical function with age. Sarcopenic status failed to detect the same impact of the L‐Kyn diet. This definition involves assessments of treadmill endurance, forelimb grip strength and hindlimb muscle mass. In our study, hindlimb muscle mass did not correlate with maximal grip strength in either sex (Pearson *r* = 0.01 and 0.16 for male and female). Future studies may want to consider in vivo assessments of muscle strength or power output in the hindlimb muscles, although we acknowledge these are more challenging and require specialised equipment. Importantly, these observations indicate the continued need for refinement of approaches to assess aging phenotypes in rodents.

Previous studies have examined aging phenotypes in MCK‐PGC1α mice [28, 29, 37, 38, 39]. Only two of these studies reported outcomes related to physical function [38, 39]. Gill et al. [38, 39] reported age‐dependent changes in outcomes like spontaneous locomotor activity, maximal running speed, running capacity and grip strength. However, these studies used only male mice, and statistical comparisons between the WT and MCK‐PGC1α mice were not reported. Nonetheless, examination of their figures suggests that MCK‐PGC1α mice had higher running capacity, which is congruent with our results. Consistent with Yang et al. [29], we also report that male, but not female, aged MCK‐PGC1α mice had greater soleus masses. Consumption of a L‐Kyn supplemented diet did not have an impact on muscle mass in the current study. Myofibre area was also larger in male MCK‐PGC1α mice in both type I and IIa myofibres when compared with WT males, consistent with Yang et al. [29]. In females, type I myofibre area was larger in MCK‐PGC1α mice, whereas type IIa myofibre areas were trending toward larger areas (*p* = 0.081) compared with WTs. Surprisingly, differences in myofibre area did not confer benefits to muscle strength in either sex, again consistent with Yang et al. [29].

Agudelo et al. [18] provided evidence that high expression of PGC1α allows skeletal muscles to utilise L‐Kyn to support the malate–aspartate shuttle leading to enhanced energetic performance. In contrast, deleting PGC1α revealed impairments in myotube oxygen consumption rates when treated with L‐Kyn. Additionally, inhibition of KATs also reduced basal and maximal myotube oxygen consumption and impaired running capacity in mice. Herein, L‐Kyn did not exacerbate age‐related declines in muscle mitochondrial respiratory function and we did not observe any benefit of L‐Kyn on muscle mitochondrial respiratory function in MCK‐PGC1α mice. It is possible that age‐dependent declines in exercise performance and muscle mitochondrial function mask the beneficial effects of L‐Kyn‐fuelled malate–aspartate shuttle‐driven mitochondrial energetics reported by Agudelo et al. in younger male mice [18]. Regardless of diet, MCK‐PGC1α male and female generally had greater muscle mitochondrial performance compared with their WT counterparts—an observation consistent with the large body of evidence on PGC1α.

The present study has some limitations. First, the intervention was performed in mice from 16 to 24 months, which represents an age range comparable to humans of ~56 to 69 years. Thus, the biological effects of elevated circulating L‐Kyn could continue to evolve in more advanced ages. We chose this time frame to avoid approaching a ‘floor effect’ where older ages increase the prevalence of frailty in all groups thereby making it more difficult to assess the impact of the diet intervention. We acknowledge that more prominent physical and physiological changes are evident at older ages in mice [25, 40]. Second, our study employed a diet model to elevate circulating L‐Kyn. This was a forced model of L‐Kyn accumulation, which may produce different biological outcomes from the age‐dependent L‐Kyn elevations. As discussed, how circulating L‐Kyn increases in aging/frailty is not fully known. Often, this increase in L‐Kyn is accompanied by lower levels of Trp. Herein, the L‐Kyn diet significantly increased circulating L‐Kyn but this did not decrease in Trp levels. Notably, consuming a Trp‐deficient diet decreases body weight and muscle mass in mice [41]. Additionally, the MCK‐PGC1α mouse line has germline overexpression that occurs from birth. This line was chosen because previous studies have demonstrated this mouse line to have enhanced kynurenine catabolism [17, 18]. Future studies may seek to employ inducible overexpression models to account for the development and potential compensatory effects of long‐term transgene expression. Finally, the mouse colony used in this study was kept in a carefully maintained vivarium, which is far from the real‐world environment of humans exposed to diverse environmental, physiological and psychological stressors over the lifespan.

In summary, this study provides evidence that elevated circulating L‐Kyn levels exacerbate declines in physical function and increase the prevalence of frailty in aging mice. Muscle‐specific overexpression of PGC1α, which enhances multiple aspects of skeletal muscle biology including L‐Kyn metabolism, counteracts some but not all of the detrimental effects of elevated L‐Kyn levels. These data support the continued investigation surrounding how aging changes the kynurenine pathway and how modulation of this pathway can alter health and wellbeing.

## Funding

This study was supported by National Institutes of Health (NIH) grant R01‐AG076490 (TER and RTH).

## Disclosure

The authors have nothing to report.

## Supporting information




**Figure S1:** Daily food consumption was not different between chow (CON) and L‐Kyn (KYN) supplemented diets. Data were analysed using two‐tailed Student's *t*‐test.
**Figure S2:** Absolute and normalised (relative to body weight) muscle and organ masses in male (A) and female (B) mice. Data were analysed using two‐way ANOVA. Mean values obtained from 14‐month‐old C57BL6 (*n* = 10/sex) obtained from the National Institute of Aging colony are shown for comparison. Data are presented as individual data points with the median.
**Figure S3:** Pearson correlation analyses between the estimated number of motor units and maximum absolute and specific forces in male (A) and female (B) mice.
**Table S1:** Primer sequences.
**Table S2:** Serum inflammatory markers in male and female mice.
**Table S3:** Post hoc pairwise comparison related to significant interactions in Figure 2.
