# Supplementary material for: Elevating Circulating L‐Kynurenine Promotes Frailty in Aging Mice
Source: J Cachexia Sarcopenia Muscle. 2026 Feb 2;17(1):e70214. doi: 10.1002/jcsm.70214 (PMC12862278; doi:10.1002/jcsm.70214)
Supplement: Supplementary file 1 — Figure S1: Daily food consumption was not different between chow (CON) and L‐Kyn (KYN) supplemented diets. Data were analysed using two‐tailed Student's t‐test. Figure S2: Absolute and normalised (relative to body weight) muscle and organ masses in male (A) and female (B) mice. Data were analysed using two‐way ANOVA. Mean values obtained from 14‐month‐old C57BL6 (n = 10/sex) obtained from the National Institute of Aging colony are shown for comparison. Data are presented as individual data points with the median. Figure S3: Pearson correlation analyses between the estimated number of motor units and maximum absolute and specific forces in male (A) and female (B) mice. Table S1: Primer sequences. Table S2: Serum inflammatory markers in male and female mice. Table S3: Post hoc pairwise comparison related to significant interactions in Figure 2. [file JCSM-17-e70214-s001.pdf]

SUPPLEMENTAL MATERIAL

Elevating Circulating L-Kynurenine Promotes Frailty in Aging Mice

Mia Y. Kawaida[1], Abigail L. Tice[1], Samuel Alvarez[1], Jacob A. Lackey[1], Benjamin Izaguirre[1], Qingping Yang[1], Lan Wei-LaPierre[1], Russell T. Hepple[2,4], Terence E. Ryan[1,3,4,#]

[1]Department of Applied Physiology and Kinesiology
[2]Department of Physical Therapy
[3]Center for Exercise Science
[4]Myology Institute
The University of Florida, Gainesville, FL, USA

Running Head: Kynurenine increase frailty in aging mice

#Correspondence: Terence E. Ryan, PhD: 1864 Stadium Rd, Gainesville, FL, 32611.
Tel: 352-294-1700 (office); email: ryant@ufl.edu; Twitter: @TerenceRyan_PhD

KEYWORDS: aging, frailty, mitochondria, muscle, physical function

Supplemental Material:

Expanded materials and methods descriptions

Supplemental Figures 1-3

Supplemental Tables 1-3

References

MATERIALS AND METHODS

Physical Function and Frailty Assessments. A phenotypic frailty assessment developed for mice (2, 3) was performed at baseline (16-mo old) prior to diet randomization and was repeated again at 20- and 24-months of age. This assessment includes analyses of body weight and composition (EchoMRI), grip strength, walking speed, treadmill endurance capacity, and voluntary wheel activity. Mice were thoroughly familiarized with the equipment and tests prior to each assessment. Additional methodological details are as follows: Bilateral forelimb grip strength was assessed using a grip strength meter (BIOSEB, BIO-GS3). Mice were gently lowered to a metal T-bar and were encouraged to firmly grip the bar. Once gripped, the tail was pulled backward horizontally while the torso remained steady until the T-bar was released. Three trials were performed with a 2-min rest between each trial, and the highest force was analyzed. Walking speed was determined using a Rotorod (Columbus Instruments, Rotamex-5). After a warm-up of 30-sec walk at 4rpm, the speed was increased by 1rmp every 8 sec to a maximum of 40rpm over a 5-min period. Walking speed was recorded as the fastest speed reached prior to inability to sustain rotation speed. Three trials were performed with a 5-min rest between each trial, and the highest walking speed was analyzed. An incremental treadmill test was performed to evaluate endurance/running capacity using an Exer-3R rodent treadmill (Columbus Instruments). Mice were run at 0% grade starting at 5m/min for 5 min, followed by 1m/min increases every 3 min until exhaustion. Mice were encouraged to continue running by light tapping with a small brush to their rump. Endurance time was recorded as the third time the mouse failed to maintain the pace

with the treadmill. Voluntary wheel activity was measured in mice that were briefly individually housed in a cage containing a running wheel for 5 days. Daily voluntary running distance was recorded (Actimetrics) and analyzed using ClockLab analysis software (Actimetrics). Obtained data on the first day were excluded from the analysis.

The physical function data above was used to assign a frailty status as follows: for physical function outcomes (running capacity, walking speed, grip strength, cage activity), a lower cut-off value of the 20th percentile was employed (4-6). For body weight, criterion values below the 20th percentile (below average body weights) and above the 80th percentile (high body weights) were used. Mice were categorized as *pre-frail* if one or two of the frailty indices met the above criteria, or *frail* if three or more of the frailty indices met the criteria. Mice that met none of the criterion scores were identified as *non-frail*.

<u>Plasma L-Kyn and Tryp Quantification</u>*.* Using a tail snip procedure, peripheral blood was collected in a heparin-coated capillary tube one week before euthanasia and centrifuged at 1,200*g* at 4°C for 10 min. Plasma was stored at -80°C until analysis. Targeted metabolomics analyses were performed by the Southeast Center for Integrated Metabolomics at the University of Florida to quantify circulating L-Kyn and Trp as previously described (8-10).

<u>*In-situ* Muscle Function</u>*.* Muscle contractile function was assessed *in-situ* using a whole animal system (Aurora Scientific Inc, Model 1300A). Mice were anesthetized with

isoflurane delivered through a nose cone using a low flow anesthesia system (Kent Scientific, SomnoSuite). After reaching a surgical plane of anesthesia, the Achilles tendon was exposed with a small incision and the plantaris tendon and the medial and then lateral portions of the gastrocnemius muscle were carefully isolated from the soleus. The distal end of the Achilles tendon was tied with a 4-0 silk suture to the lever arm of the force transducer. The sciatic nerve was isolated and stimulated at 2mA via bipolar electrodes using square-wave pulses (Aurora Scientific, model 701A stimulator) to elicit muscle contractions. Lab-View–based DMC program (Aurora Scientific Inc, version 615A.v6.0) was used for data collection and servomotor control. Muscle function testing was performed on a temperature-controlled platform to maintain a body temperature of 37°C. First, optimal length was obtained via twitch contractions. Next, isometric contractions were elicited at stimulation frequencies of 1Hz, 20Hz, 50Hz, and 150Hz (0.2ms pulse width and 0.5s train duration) with a 1-min rest between contractions. The peak tetanic force was reported as absolute force and specific force (absolute force normalized to muscle mass). Isotonic power output was determined by performing an after-loaded contraction hich involve electrically stimulating the nerve with supramaximal voltage (2 mA; 0.2-ms pulse width; 250-ms train duration) at 150 Hz to activate all motor units and allowing the muscle to shorten once it surpassed 35% of the 150-Hz peak isometric force. Muscle shortening velocity was calculated as the change in distance (millimeters) from a 10-ms period which began 20 ms after the initial length change. Power output was calculated by multiplying shortening velocity by the corresponding force output. Muscle fatigability was tested by completing 150 repetitive tetanic contractions at 100Hz (0.25ms pulse width and 0.5s train duration) every two

seconds. Motor unit number estimation (MUNE) was performed according to the protocol previously described (11).

Muscle Histology. Soleus muscle was carefully dissected, embedded on a cryomold with optimal cutting temperature compound, and frozen in liquid nitrogen-cooled isopentane. Transverse sections were cut from the muscle at 10μm using a Leica 3050S and mounted on microscope slides. All muscle sections were then blocked at room temperature for one hour in 1x phosphate-buffered saline (PBS) containing 5% goat serum and 1% bovine serum albumin. Muscle sections were incubated overnight at 4°C with primary antibodies for laminin (Millipore-Sigma, Cat. No. L9393; 1:200 dilution), myosin heavy chain type I (DSHB, Cat. No. BA-D5; 1:100 dilution), and myosin heavy chain type IIA (DSHB, Cat. No. SC-71; 1:100 dilution). The following morning, four of 5-min washes with 1x PBS were performed, before the sections were incubated with secondary antibodies: Alexa flour 647 goat anti-rabbit immunoglobulin G (ThermoFisher Scientific, Cat. No. A21245; 1:500 dilution), Alexa Fluor 555 goat anti-mouse immunoglobulin G2b (ThermoFisher Scientific, Cat. No. A21147; 1:250 dilution), and Alexa Fluor 488 goat anti-mouse immunoglobulin G1 (ThermoFisher Scientific, Cat. No. A21121; 1:250 dilution) for 90 minutes. After four washes with 1x PBS, coverslips were mounted onto the slides using Vectashield hardmount (Vector Laboratories, Cat. No. H-1500). Slides were imaged at 20× magnification with an Evos FL2 Auto microscope (ThermoFisher Scientific), and tiled images of the entire muscle section were obtained for analysis. Quantification of myofiber cross-sectional area (CSA) and type I and IIA

fibers were performed using Muscle J2 (12), an automated analysis software developed in Fiji.

Mitochondrial isolation. Mitochondria were isolated from the quadriceps and gluteus muscles as previously described (8). Briefly, the dissected muscles were placed in ice cold mitochondrial isolation medium (MIM; 50mM MOPS, 100mM KCl, 1mM EGTA, 5mM $MgSO_4$), followed by three minutes of mincing. The minced muscles were incubated in MIM containing 0.025% w/v trypsin for three minutes and centrifuged at 500xG for five minutes at 4°C. The resulting supernatant was discarded, and the remained pellet was resuspended with MIM containing 0.02% w/v bovine serum albumin (BSA). The sample was then homogenized using a glass-Teflon homogenizer and subsequently centrifuged at 800xG for ten minutes at 4°C. The resulting supernatant was collected and centrifuged at 10,000xG for ten minutes at 4°C to create a mitochondrial rich pellet. The pellet was washed with MIM to remove damaged mitochondria and was then gently resuspended in MIM. Protein concentration of the final mitochondrial resuspension was determined using bicinchoninic acid protein assay kit (ThermoFisher Scientific, Cat. No. A53225).

High-resolution respirometry. As previously described (13), an Oroboros Oxygraph-2k (O2K) was used to measure oxygen consumption (JO_2) at 37°C in buffer D (105mM K-MES, 30mM KCl, 1mM EGTA, 10mM K_2HPO_4, 5mM $MgCl_2$-$6H_2O$, 2.5mg/mL BSA, pH 7.2) supplemented with 5mM creatine (Cr). To assess oxidative phosphorylation

(OXPHOS), 20-30µg of mitochondria were loaded into the O2K chamber and energized using 5mM pyruvate + 2.5mM malate (carbohydrate) or 0.02mM palmityolcarnitine + 2.5mM malate (fatty acid). Next, we employed a creatine kinase (CK) clamp system to control the extramitochondrial ATP/ADP ratio (ΔG_{ATP}) as previously described (14). The integrity of the outer mitochondrial membrane was assessed via the addition of 0.005mM cytochrome c to assess the integrity of the outer mitochondrial membrane. Samples with greater than 30% increase in respiration following cytochrome c were excluded from the analysis. The OXPHOS conductance was calculated as the slope of the relationship between JO_2 and ΔG_{ATP}.

Mitochondrial membrane potential. Mitochondrial membrane potential ($\Delta\Psi$) was fluorometrically assessed in buffer D containing 20µg of mitochondria, 5mM Cr, 0.0002mM fluorescent cationic dye tetramethylrhodamine methyl ester (TMRM), 1mM phosphocreatine (PCr), and 20U/mL CK. Mitochondrial respiration was initiated with 5mM ATP and either 5mM pyruvate + 2.5mM malate or 0.02mM palmityolcarnitine + 2.5mM malate. Then, 1mM PCr was sequentially added to employ the creatine kinase clamp. With emission setting of 590nm, TMRM was exited both at 572nm and 551nm, and the ratio of 572:590/551:590 was converted to mV by a standard curve. Fluorometric assays were performed with a QuantaMaster Spectrofluorometer (Horiba Scientific, QM-400).

Mitochondrial H_2O_2 production. Production of H_2O_2 in the isolated skeletal muscle mitochondria was measured fluorometrically via Amplex UltraRed (AUR)/horseradish peroxidase (HRP) detection system (excitation/emission 530:590nm) as previously described (15). Briefly, 10μg of mitochondria were added to buffer D supplemented with 5mM Cr, 20U/mL CK, 10μM AUR, 1U/mL HRP, 20U/mL superoxide dismutase (SOD), 5mM ATP, and 0.1μM auranofin which inhibits endogenous thioredoxin reductase, allowing a more accurate assessment of H_2O_2 production (14). Mitochondria were energized with either 5mM pyruvate + 2.5mM malate or 0.02mM palmityolcarnitine + 2.5mM malate. Using the CK clamp, experiments were performed at two levels of energy demands: (a) state 2 maximal H_2O_2 production with no addition of PCr and (b) resting ΔG_{ATP} (–64.6kJ/mol) induced by adding 30mM PCr. A total reaction volume of 220μL was read for 30 minutes using a BioTek Synergy 2 Multimode Microplate Reader at 37°C. Fluorescence values were converted to pmoles of H_2O_2 using a standard curve.

RNA Isolation and Quantitative PCR. Total RNA was extracted from the gastrocnemius muscle using TRIzol (Invitrogen, Cat. No. 15-596-018) for lysis and Direct-zol RNA MiniPrep kit (Zymo Research, Cat. No. R2052) following the manufacturer's protocol. Using the LunaScript RT Supermix kit (New England Biolabs, Cat. No. E3010L), cDNA was reverse transcribed from 500ng of total RNA. Quantitative PCR (qPCR) was performed on a Quantstudio 3 (ThermoFisher Scientific) using Luna Universal qPCR master mix for Sybr Green primers (New England Biolabs, Cat. No. M3003X). All primers used in this analysis are described in Supplemental Table 1, and *L32* was used

as the housekeeping gene. Relative gene expression was calculated using $2^{-\Delta\Delta CT}$ from WT-CON group.

Serum cytokine/chemokine analysis. At euthanasia, blood was collected via cardiac puncture, stored at room temperature for one hour to allow the blood to clot, and centrifuged at 1,000*g* for 10 min. Serum was stored at -80°C until analysis. A multiplex analysis was performed by Eve Technologies (Calgary, AB, Canada) using the Mouse Cytokine 32-PlexDiscovery Assay Array (M32) and the Luminex 200 system. The 32 targets included Eotaxin, G-CSF, GM-CSF, IFNγ, IL-1α, IL-1β, IL-2, IL-3, IL-4, IL-5, IL-6, IL-7, IL-9, IL-10, IL-12p40, IL-12p70, IL-13, IL-15, IL-17A, IP-10, KC, LIF, LIX, MCP-1, M-CSF, MIG, MIP-1α, MIP-1β, MIP-2, RANTES, TNFα, and VEGF. Any cytokine with a sample size of less than 5 was excluded from the analysis for appropriate statistical tests.

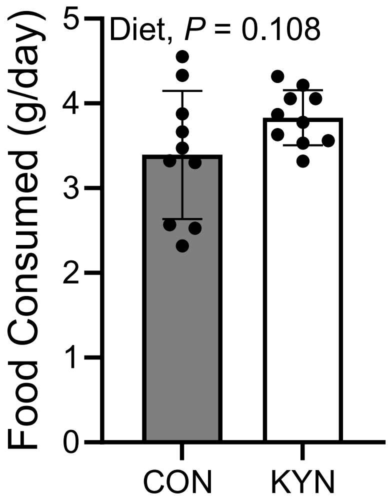

<u>Supplemental Figure 1.</u> Daily food consumption was not different between chow (CON) and L-Kyn (KYN) supplemented diets. Data were analyzed using two-tailed Student's *t*-test.

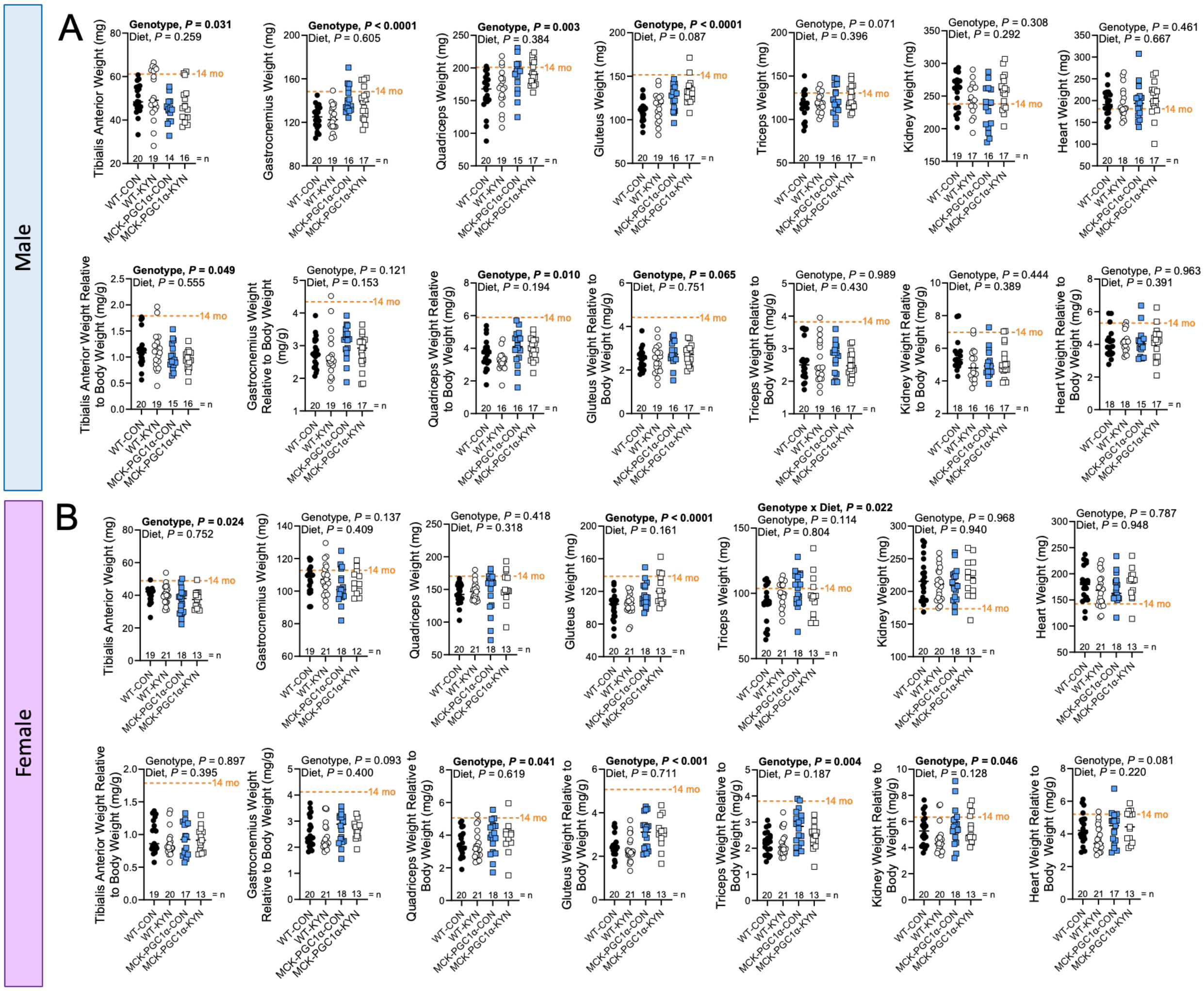

Supplemental Figure 2. Absolute and normalized (relative to body weight) muscle and organ masses in male (A) and female (B) mice. Data were analyzed using two-way ANOVA. Mean values obtained from 14 mo. old C57BL6 (n=10/sex) obtained from the National Institute of Aging colony are shown for comparison. Data are presented as individual data points with the median.

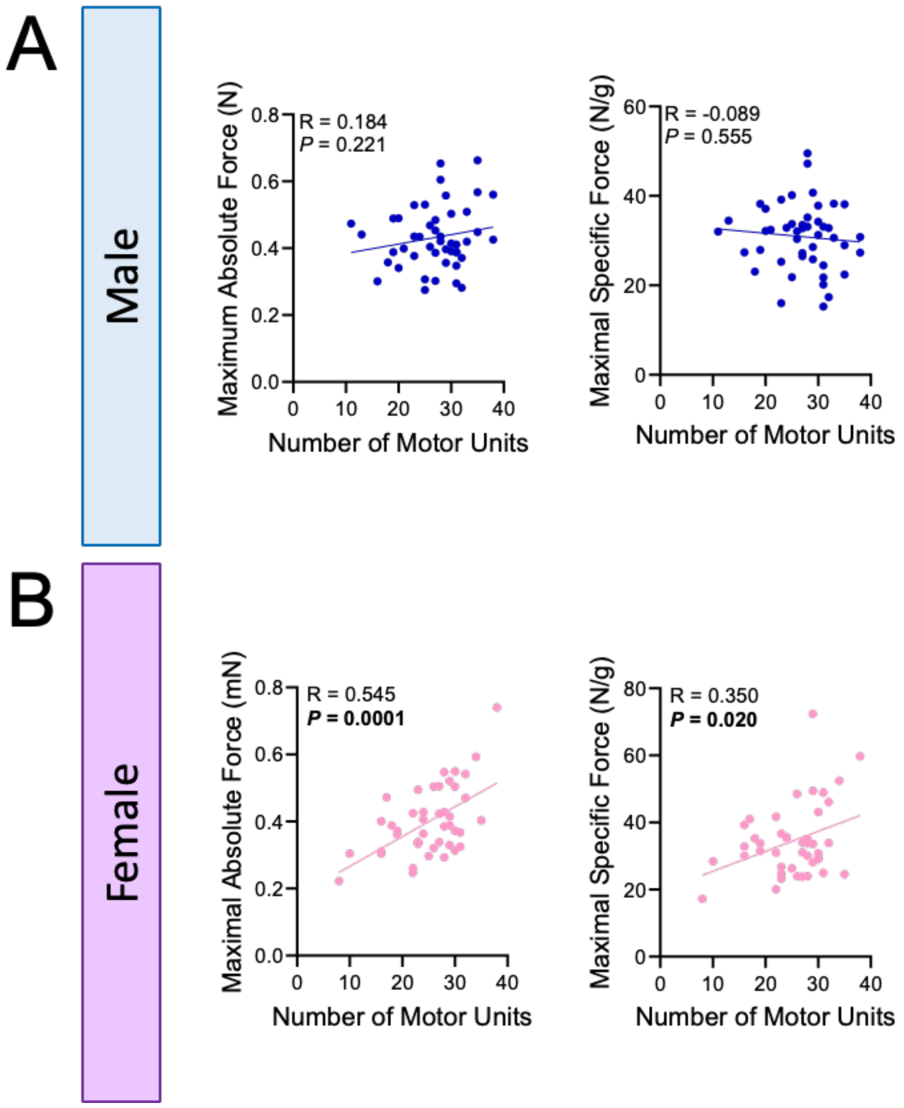

Supplemental Figure 3. Pearson correlation analyses between the estimated number of motor units and maximum absolute and specific forces in male (A) and female (B) mice.

Supplemental Table 1. Primer sequences.

Species	Gene	Primer sequences (5' → 3')
Mus Musculus	*Ccbl1* (KAT1)	
	Forward	TCATGCTCAACCAGTACACC
	Reverse	GTCACCAGCACATTCTTGAGT
Mus Musculus	*Ccbl2* (KAT3)	
	Forward	CACGACACTCTGTGCATCAG
	Reverse	GTCTTGCCAGCACTTCCTAT
Mus Musculus	*Ctsl1*	
	Forward	GTGGACTGTTCTCACGCTCAAG
	Reverse	TCCGTCCTTCGCTTCATAGG
Mus Musculus	*Gabapral1*	
	Forward	CATCGTGGAGAAGGCTCCTA
	Reverse	ATACAGCTGGCCCATGGTAG
Mus Musculus	*Got2* (KAT4)	
	Forward	GTATTCCAACCCACCTCTCAA
	Reverse	GCCATGCCTTTCACCTCTT
Mus Musculus	*L32*	
	Forward	TTCCTGGTCCACAATGTCAA
	Reverse	GGCTTTTCGGTTCTTAGAGGA
Mus Musculus	*Ndufa5*	
	Forward	AGCTGGATATGGTCAAGGCG
	Reverse	GCCACTTCCACTGGTTAGCA
Mus Musculus	*Ppargc1a*	
	Forward	TATGGAGTGACATAGAGTGTGCT
	Reverse	CCACTTCAATCCACCCAGAAAG

Supplemental Table 2. Serum inflammatory markers in male and female mice.

Inflammatory markers		WT-CON	WT-KYN	MCK-PGC1α-CON	MCK-PGC1α-KYN	Kruskal-Wallis *P* value
Eotaxin	Male	435.9 ± 211.7	525.9 ± 88.88	560.4 ± 259.8	355.9 ± 205.3	0.32
	Female	553.0 ± 214.6	524.3 ± 276.7	599.6 ± 319.7	736.5 ± 455.4	0.67
G-CSF	Male	610.6 ± 419.1	403.5 ± 122.5	455.7 ± 312.1	286.0 ± 116.2	0.33
	Female	710.4 ± 555.2	679.5 ± 398.9	461.8 ± 250.4	480.7 ± 216.2	0.60
GM-CSF	Male	47.7 ± 42.53	10.19 ± 4.12	33.57 ± 22.64	39.03 ± 35.76	0.14
	Female	51.15 ± 35.60	27.03 ± 26.79	22.60 ± 14.86	17.92 ± 14.62	0.30
IL-1 α	Male	2094 ± 1568	1115 ± 656.8	1893 ± 1604	1367 ± 778.3	0.80
	Female	529.9 ± 302.0	507.1 ± 432.3	1085 ± 793.2	779.0 ± 521.7	0.29
IL-2	Male	12.88 ± 8.39	6.83 ± 7.15	53.34 ± 49.36	12.93 ± 5.08	0.45
	Female	Number of samples within the detectable limits not sufficient for statistical analysis				
IL-5	Male	4.85 ± 2.43	3.28 ± 1.42	4.76 ± 2.80	3.81 ± 2.78	0.72
	Female	5.06 ± 2.16	4.44 ± 1.34	10.19 ± 6.31	3.98 ± 2.13[a]	**0.05**
IL-6	Male	79.90 ± 61.56	55.61 ± 36.43	65.63 ± 51.26	117.9 ± 83.99	0.45
	Female	64.69 ± 24.00	40.81 ± 30.39	67.70 ± 30.99	55.70 ± 53.62	0.35
IL-7	Male	18.57 ± 17.27	11.47 ± 11.48[a]	112.7 ± 95.82[b]	38.29 ± 32.68[b]	**0.02**
	Female	Number of samples within the detectable limits not sufficient for statistical analysis				
IL-10	Male	25.90 ± 11.85	10.82 ± 4.88	27.27 ± 13.64	75.22 ± 111.4	**0.03**

	Female	45.00 ± 19.05	58.97 ± 29.56	37.19 ± 26.59	23.23 ± 6.49[b]	**0.08**
IL-20p40	Male	37.24 ± 43.87	20.12 ± 16.96	109.7 ± 172.2	15.29 ± 6.49	0.62
	Female	22.14 ± 12.39	20.39 ± 12.32	8.52 ± 2.20[b]	11.58 ± 8.97	**0.08**
IL-20p70	Male	10.15 ± 6.82	12.45 ± 9.44	55.64 ± 64.31	25.42 ± 31.39	0.12
	Female	16.68 ± 12.81	41.18 ± 57.52	55.56 ± 56.96	17.00 ± 18.39	0.32
IL-13	Male	383.7 ± 588.0	26.92 ± 5.90	85.15 ± 90.55	60.58 ± 63.70	0.57
	Female	30.41 ± 11.89	85.43 ± 74.73	27.04 ± 11.81	37.03 ± 14.11	0.24
IL-15	Male	28.81 ± 11.01	33.83 ± 25.01	419.6 ± 459.3[b]	283.7 ± 302.8	**0.03**
	Female	41.16 ± 14.31	72.60 ± 44.48	48.03 ± 24.26	66.29 ± 49.67	0.61
IL-17	Male	3.08 ± 1.46	2.88 ± 1.33	4.80 ± 3.46	5.14 ± 3.89	0.80
	Female	2.29 ± 0.82	5.14 ± 3.04	4.22 ± 2.99	3.50 ± 2.65	0.26
IP-10	Male	66.50 ± 30.26	54.30 ± 11.95	78.44 ± 27.84	65.15 ± 40.53	0.39
	Female	66.39 ± 19.23	97.65 ± 32.52	78.14 ± 20.86	93.64 ± 35.50	0.22
KC	Male	155.9 ± 116.7	113.6 ± 33.43	144.9 ± 74.95	124.2 ± 63.44	0.77
	Female	167.3 ± 90.29	120.8 ± 88.36	203.6 ± 145.0	242.9 ± 140.2	0.33
LIX	Male	3360 ± 1613	5972 ± 1021	5269 ± 3124	3901 ± 1970	0.17
	Female	4585 ± 2089	3191 ± 1885	4767 ± 842.8	4786 ± 2442	0.28
M-C SF	Male	12.66 ± 18.39	6.51 ± 6.73	40.52 ± 64.14	1.84 ± 2.08	0.19
	Female	4.83 ± 4.70	2.61 ± 0.82	2.19 ± 1.33	2.14 ± 1.11	0.62
MCP-1	Male	81.30 ± 43.17	85.39 ± 40.80	239.1 ± 285.2	130.1 ± 125.8	0.91
	Female	78.73 ± 50.46	105.5 ± 62.88	122.2 ± 69.38	156.2 ± 121.7	0.36

MIG	Male	763.7 ± 417.3	1138 ± 441.1	1161 ± 478.1	1041 ± 727.1	0.26
	Female	1084 ± 316.2	1835 ± 802.3	1304 ± 591.4	1536 ± 828.7	0.15
MIP-1α	Male	162.6 ± 136.9	55.11 ± 23.03	132.5 ± 100.4	105.7 ± 96.23	0.43
	Female	75.42 ± 45.96	55.73 ± 46.00	70.87 ± 40.42	23.67 ± 8.96[a]	**0.08**
MIP-1β	Male	225.3 ± 98.06	98.01 ± 75.62[a]	156.8 ± 65.48	152.5 ± 112.4	**0.09**
	Female	197.0 ± 119.5	192.9 ± 106.5	126.0 ± 53.40	82.03 ± 47.15[b]	**0.04**
MIP-2	Male	87.63 ± 68.90	119.0 ± 38.52	186.7 ± 154.4	105.7 ± 73.57	0.23
	Female	85.76 ± 42.62	74.58 ± 44.17	62.74 ± 41.80	86.91 ± 44.15	0.56
RANTES	Male	62.98 ± 41.76	31.48 ± 11.12	50.28 ± 33.04	36.58 ± 27.24	0.39
	Female	28.42 ± 10.37	55.97 ± 48.10	36.82 ± 17.12	30.12 ± 13.91	0.51
TNFα	Male	21.33 ± 26.02	8.00 ± 6.26	91.69 ± 241.0	8.24 ± 6.80	0.90
	Female	6.36 ± 3.86	7.13 ± 3.71	7.05 ± 3.67	16.83 ± 15.06	0.63

[a]*P*<0.05 for posthoc diet effect (within genotype); [b]*P*<0.05 for posthoc genotype effect (within diet). Posthoc analyses done using Benjamini and Hochberg false discovery rate.

Supplemental Table 3. Post-hoc pairwise comparison related to significant interactions in Figure 2.

Outcome Measure	Male	
	Pairwise Comparison	Individual *P*-Value
Male Treadmill Running Capacity	16 mo:WT-CON vs. 16 mo:MCK-PGC1α-CON	0.0318
	16 mo:WT-KYN vs. 20 mo:WT-CON	0.0469
	16 mo:WT-KYN vs. 20 mo:WT-KYN	0.0010
	16 mo:WT-KYN vs. 20 mo:MCK-PGC1α-KYN	0.0049
	20 mo:WT-CON vs. 20 mo:MCK-PGC1α-CON	0.0290
	20 mo:MCK-PGC1α-CON vs. 20 mo:MCK-PGC1α-KYN	0.0023
	24 mo:WT-CON vs. 24 mo:WT-KYN	0.0259
	24 mo:MCK-PGC1α-CON vs. 24 mo:MCK-PGC1α-KYN	0.0732
Cage Activity	20 mo:WT-CON vs. 20 mo:MCK-PGC1α-CON	0.0443
	20 mo:WT-KYN vs. 20 mo:MCK-PGC1α-CON	0.0232
	20 mo:MCK-PGC1α-CON vs. 20 mo:MCK-PGC1α-KYN	0.0045
	24 mo:MCK-PGC1α-CON vs. 24 mo:MCK-PGC1α-KYN	0.0775
	24 mo:WT-CON vs. 24 mo:WT-KYN	0.080

REFERENCES

1. Lin J, Wu H, Tarr PT, Zhang CY, Wu Z, Boss O, et al. Transcriptional co-activator PGC-1 alpha drives the formation of slow-twitch muscle fibres. *Nature.* 2002;418(6899):797-801.
2. Baumann CW, Kwak D, and Thompson LV. Assessing onset, prevalence and survival in mice using a frailty phenotype. *Aging (Albany NY).* 2018;10(12):4042-53.
3. Baumann CW, Kwak D, and Thompson LV. Phenotypic Frailty Assessment in Mice: Development, Discoveries, and Experimental Considerations. *Physiology (Bethesda).* 2020;35(6):405-14.
4. Baumann CW, Kwak D, and Thompson LV. Assessing onset, prevalence and survival in mice using a frailty phenotype. *Aging-Us.* 2018;10(12):4042-53.
5. Baumann CW, Kwak D, and Thompson LV. Phenotypic Frailty Assessment in Mice: Development, Discoveries, and Experimental Considerations. *Physiology.* 2020;35(6):405-14.
6. Kwak D, Baumann CW, and Thompson LV. Identifying Characteristics of Frailty in Female Mice Using a Phenotype Assessment Tool. *J Gerontol a-Biol.* 2020;75(4):640-6.
7. Kerr HL, Krumm K, Anderson B, Christiani A, Strait L, Li T, et al. Mouse sarcopenia model reveals sex- and age-specific differences in phenotypic and molecular characteristics. *J Clin Invest.* 2024;134(16).
8. Balestrieri N, Palzkill V, Pass C, Tan J, Salyers ZR, Moparthy C, et al. Activation of the Aryl Hydrocarbon Receptor in Muscle Exacerbates Ischemic Pathology in Chronic Kidney Disease. *Circulation research.* 2023;133(2):158-76.
9. Thome T, Vugman NA, Stone LE, Wimberly K, Scali ST, and Ryan TE. A tryptophan-derived uremic metabolite-Ahr-Pdk4 axis governs skeletal muscle mitochondrial energetics in chronic kidney disease. *JCI Insight.* 2024.
10. Thome T, Kumar RA, Burke SK, Khattri RB, Salyers ZR, Kelley RC, et al. Impaired muscle mitochondrial energetics is associated with uremic metabolite accumulation in chronic kidney disease. *Jci Insight.* 2021;6(1).
11. Zhong R, Dionela DLA, Kim NH, Harris EN, Geisler JG, and Wei-LaPierre L. Micro-Doses of DNP Preserve Motor and Muscle Function with a Period of Functional Recovery in Amyotrophic Lateral Sclerosis Mice. *Ann Neurol.* 2024.
12. Danckaert A, Trignol A, Le Loher G, Loubens S, Staels B, Duez H, et al. MuscleJ2: a rebuilding of MuscleJ with new features for high-content analysis of skeletal muscle immunofluorescence slides. *Skelet Muscle.* 2023;13(1):14.
13. Thome T, Salyers ZR, Kumar RA, Hahn D, Berru FN, Ferreira LF, et al. Uremic metabolites impair skeletal muscle mitochondrial energetics through disruption of the electron transport system and matrix dehydrogenase activity. *Am J Physiol Cell Physiol.* 2019;317(4):C701-C13.
14. Fisher-Wellman KH, Davidson MT, Narowski TM, Lin CT, Koves TR, and Muoio DM. Mitochondrial Diagnostics: A Multiplexed Assay Platform for Comprehensive Assessment of Mitochondrial Energy Fluxes. *Cell Rep.* 2018;24(13):3593-606.e10.

15. Thome T, Kumar RA, Burke SK, Khattri RB, Salyers ZR, Kelley RC, et al. Impaired muscle mitochondrial energetics is associated with uremic metabolite accumulation in chronic kidney disease. *JCI Insight*. 2020;6(1).
